# The relationship between neighborhood economic deprivation and community-acquired pneumonia related admissions in Maryland

**DOI:** 10.3389/fpubh.2024.1412671

**Published:** 2024-07-18

**Authors:** Oluwasegun Akinyemi, Mojisola Fasokun, Eunice Odusanya, Terhas Weldeslase, Ofure Omokhodion, Miriam Michael, Kakra Hughes

**Affiliations:** ^1^Department of Surgery Outcomes Research Center, Howard University College of Medicine, Washington, DC, United States; ^2^Department of Health Policy and Management, University of Maryland, College Park, MD, United States; ^3^Department of Epidemiology, University of Alabama at Birmingham, Birmingham, AL, United States; ^4^Department of Surgery, Howard University College of Medicine, Washington, DC, United States; ^5^Department of Epidemiology, John Hopkins University Bloomberg School of Public Health, Baltimore, MD, United States; ^6^Department of Internal Medicine, Howard University College of Medicine, Washington, DC, United States

**Keywords:** distressed community index (DCI), community-acquired pneumonia (CAP), health disparities, socioeconomic determinants of health, neighborhood economic deprivation, population health

## Abstract

**Introduction:**

Community-acquired pneumonia (CAP) is a major health concern in the United States (US), with its incidence, severity, and outcomes influenced by social determinants of health, including socioeconomic status. The impact of neighborhood socioeconomic status, as measured by the Distressed Communities Index (DCI), on CAP-related admissions remains understudied in the literature.

**Objective:**

To determine the independent association between DCI and CAP-related admissions in Maryland.

**Methods:**

We conducted a retrospective study using the Maryland State Inpatient Database (SID) to collate data on CAP-related admissions from January 2018 to December 2020. The study included adults aged 18–85 years. We explored the independent association between community-level economic deprivation based on DCI quintiles and CAP-related admissions, adjusting for significant covariates.

**Results:**

In the study period, 61,467 cases of CAP-related admissions were identified. The patients were predominantly White (49.7%) and female (52.4%), with 48.6% being over 65 years old. A substantive association was found between the DCI and CAP-related admissions. Compared to prosperous neighborhoods, patients living in economically deprived communities had 43% increased odds of CAP-related admissions.

**Conclusion:**

Residents of the poorest neighborhoods in Maryland have the highest risk of CAP-related admissions, emphasizing the need to develop effective public health strategies beneficial to the at-risk patient population.

## Introduction

Community-acquired pneumonia (CAP) is consistently among the leading causes of morbidity and mortality worldwide, with substantial clinical and economic impact (1, 2). The disease occurs in approximately 4 million adults in the United States (US), accounting for about 1.5 million hospitalizations (3, 4). Among hospitalized adults, CAP is associated with a substantial mortality burden of over 100,000 in-hospital deaths per year (3, 5). The combination of CAP and influenza remains among the top 10 leading causes of death in the US, ranking 9th in 2020, while the mortality rate of CAP ranks the highest among infectious diseases (5–8).

CAP therefore represents a significant public health challenge, imposing a considerable burden on individuals and the healthcare system (3, 5, 9). The association between socioeconomic factors, environmental influences, and the development of infectious diseases, such as CAP, is increasingly recognized as a critical determinant of health outcomes (10–12).

The pathophysiology of CAP involves complex microbial mechanisms, majorly influenced by bacterial pathogens such as *Streptococcus pneumoniae*, *Haemophilus influenzae*, and atypical organisms like *Mycoplasma pneumoniae* (7, 13, 14). The immune response to these pathogens is mediated by various cellular processes, including the activation of alveolar macrophages and the release of inflammatory cytokines such as tumor necrosis factor-alpha (TNF-α), interleukin-1 (IL-1), and interleukin-6 (IL-6) (15, 16). These cytokines contribute to characteristic pneumonia symptoms, like fever, cough, and chest pain. TNF-α, especially, has been identified as an important cytokine, responsible for regulating several cellular activities that lead to pneumonia’s clinical manifestations (17–19). It promotes the recruitment of neutrophils, increased vascular permeability, and consolidation of lung tissues. Targeting the inflammatory cascade provides a potential therapeutic approach, given its significant role in the exacerbation of pneumonia symptoms (19).

Considering the environment and its condition further expands the gap in patient outcomes associated with CAP. The prevalence of respiratory infections in recent years has been closely linked to the degradation of natural ecosystems through pollution (20–23). Allergens and particulate matter, which serve as pollutants, impair immune responses, making individuals more susceptible to respiratory infections like CAP (24, 25). Similarly, climate change plays a pivotal role in the proliferation of infectious agents and increased frequency of extreme weather events, potentially worsening the disease burden of CAP (26, 27). Notably, these environmental factors intertwine with neighborhood economic deprivation to a large extent (23, 26).

While CAP is a crucial health concern, it is also a substantial economic burden in the US. With the steady increase in the CAP-related cases, the US spends a large amount of the healthcare expenditures on CAP, accounting for both its direct and indirect costs (28–31). Direct costs such as medical expenses associated with doctor’s visits, medications, and hospitalizations are covered by revenue generated from American taxpayers (31, 32). Indirect costs which entail decreased job performance and productivity losses due to absenteeism are a significant portion of this financial burden (31). In total, the annual expenditure for pneumonia events in 2014 was $3 billion for individuals with diabetes and $9 billion for those without diabetes in the United States (32). This high economic burden of CAP can likely be attributed to each patient’s socioeconomic position (33).

The socioeconomic context that defines an individual significantly affect their health outcomes (34). Neighborhoods characterized by economic deprivation often experience adverse environmental hazards, coupled with limited healthcare access, and insufficient resources for disease management (35). Investigating the critical interplay between neighborhood economic deprivation and CAP-related hospital admissions, this study aims to determine their independent association in the state of Maryland using the Distressed Community Index (DCI). We seek to provide valuable insights into geographical variations and emphasize the urgency of addressing health disparities.

## Methods

### Study design and dataset

In this present study, we conducted a retrospective analysis of all hospitalizations with the diagnosis of CAP in the Maryland State Inpatient Databases (SID) from January 2018 to December 2020. The SIDs, as designed by the Healthcare Cost and Utilization Project (HCUP), are state-specific inpatient department databases that contain information on all hospital visits that result in admission (36). These databases are a valuable resource for examining healthcare utilization, access, charges, quality, and outcomes. They are also useful for analyzing rare conditions due to its extensive sample size and each of the reported discharges are de-identified.

Using the Maryland SID, we utilized the DCI to measure community-level socioeconomic deprivation. We further performed multivariate logistic regression analyses to examine the independent associations between neighborhood socioeconomic factors that influence CAP-related admissions.

### Inclusion and exclusion criteria

The inclusion criteria were defined as all patients residing in Maryland who underwent hospitalization following a primary CAP diagnosis between January 2018 and December 2020. Exclusion criteria included individuals younger than 18 years or older than 85 years, as well as patients lacking complete zip code information.

### Dependent variable

The primary outcome of this study was the occurrence of CAP-related admissions. These admissions were identified based on the International Classification of Diseases-10 (ICD-10) diagnosis codes and records (see Supplementary file).

### Independent variable

The variable of interest in this analysis was the DCI. We utilized the DCI as a tool to measure community-level socioeconomic deprivation. The Economic Innovation group created the DCI using seven local metrics to quantify socioeconomic risk (37). These metrics include the proportion of the population (age ≥ 25 years) without a high school diploma or equivalent, the ratio of housing units that are vacant after adjustment for recreational, seasonal, or occasional use vacancies, and the proportion of the population age 25–54 years who are not working (either unemployed or not in the labor force). Others include the proportion of residents living below the federal poverty rate, the median household income as a percent of a metro area or the state median household income, and changes in the number of employees working in the area and the number of business establishments in the zip code. From this information, scores ranging from 0 (no distress) to 100 (severe distress) are obtained by ranking ZIP codes on each of those metrics, averaging them, and normalizing data to generate a relative measure of socioeconomic distress. These scores are further stratified to classify American communities into five levels of socioeconomic distress (prosperous, comfortable, mid-tier, at-risk, and distressed) (37). This meticulous approach allows the DCI to provide a nuanced view of the socioeconomic landscapes across various communities, aiding policymakers, researchers, and the public in identifying areas in need of attention and resources (38).

### Covariates

Our analysis included significant covariates to address potential confounders and better isolate the independent association between neighborhood poverty and CAP-related admissions. Demographic data assessed were age (18–45 years, 45–65 years, and > 65 years), sex (male, females), insurance type (Medicare, Medicaid, Private Insurance, Self-pay and Other), and estimated median household income of residents in the patient’s ZIP codes stratified into 4 quartiles (quartile I: $1 - $49,999, quartile II: $50,000 - $64,999, quartile III: $65,000 - $85,999 and quartile IV: $86,000+). We also utilized the HCUP classification for patients’ race as Non-Hispanic White, Non-Hispanic Black, Hispanic, Non-Hispanic Asian/Pacific Islander, Non-Hispanic Native American, and Non-Hispanic Others (36). We identified patients with some traditional risk factors (preexisting diabetes mellitus, hypertension, obesity, dementia, and HIV infection) and included patients with some lifestyle behaviors (alcohol addiction and smoking) in the analysis (see Supplementary file).

### Statistical analysis

We utilized descriptive statistics such as frequencies and percentages to describe patients’ demographics, common comorbidities, and lifestyle behaviors. Using Pearson chi-square tests, we evaluated the relationship between the studied variables and CAP-related admissions. In the final multivariate logistic regression analyses, we estimated the independent association between DCI and the risk of hospitalizations because of CAP. The results were reported as adjusted odds ratios and 95% confidence intervals. A 2-tailed *p*-value <0.05 was considered statistically significant. All statistical analyses were performed using the STATA 14 (StataCorp College Station, TX).

## Results

There were 1,695,697 hospitalizations reported in the Maryland SID during the study period, of which 61,467 were CAP-related admissions.

Table 1 presents the baseline characteristics of the patient population, stratified by indications for admission (CAP vs. No CAP). Age distribution showed significant differences (*p* < 0.001) between the two groups. Hospitalized patients with CAP were older, with 48.6% over 65 years compared to 33.9% in the No CAP group. Additionally, the 18-45 years age group constituted only 16.2% of the CAP patients versus 40.5% of the No CAP patients. Gender distribution also varied significantly (*p* < 0.001) as a higher percentage of females were in the CAP group (52.4%) compared to the No CAP group (43.0%).

**Table 1:** Baseline distribution of study variables stratified by indications for admissions related to community acquired pneumonia, 2018–20.

Variable	Total	CAP	No CAP	*p* value
	(*n* = 1,695,697)	(*n* = 61,467)	(*n* = 1,634,230)	
Age (Years)				<0.001
18–45	672,405 (39.7%)	9,926 (16.2%)	662,479 (40.5%)	
45–65	439,880 (25.9%)	21,682 (35.3%)	418,198 (25.6%)	
> 65	583,412 (34.4%)	29,859 (48.6%)	553,553 (33.9%)	
Female	961,422 (56.7%)	32,186 (52.4%)	701,891 (43.0%)	<0.001
Race/ethnicity				<0.001
White	891,998 (33.5%)	30,084 (49.7%)	861,914 (53.4%)	
Black	561,792 (44.5%)	21,796 (36.0%)	539,996 (33.4%)	
Hispanics	123,753 (7.4%)	5,536 (9.1%)	118,217 (7.3%)	
Asian/Pacific Islander	49,991 (3.0%)	1,464 (2.4%)	48,527(3.0%)	
Native Americans	3,168 (0.2%)	147 (0.2%)	3,021 (0.2%)	
Others	44,780 (2.7%)	1,519 (2.5%)	43,261 (2.7%)	
Insurance				<0.001
Medicare	671,648 (40.9%)	32,956 (55.4%)	638,692 (40.3%)	
Medicaid	425,407 (25.9%)	11,095 (18.7%)	414,312 (26.1%)	
Private	517,787 (31.5%)	13,959 (23.5%)	503,828 (31.8%)	
Uninsured	25,523 (1.6%)	1,396 (2.4%)	24,127 (1.5%)	
Others	3,942 (0.2%)	64 (0.1%)	3,878 (0.2%)	
Income				<0.001
Quartile I	206,598(12.3%)	7,991 (13.1%)	198,607 (12.3%)	
Quartile II	221,673 (13.2%)	7,804 (12.8%)	213,869 (13.2%)	
Quartile III	524,801 (31.2%)	19,499 (32.0%)	505,302 (31.2%)	
Quartile IV	728,257 (43.3%)	25,671 (42.1%)	702,586 (43.4%)	
DCI				<0.001
Prosperous	392,397 (25.5%)	12,987 (23.1%)	379,410 (25.6%)	
Comfortable	397,856 (25.9%)	14,340 (25.5%)	383,516 (25.9%)	
Mid-tier	347,640 (22.6%)	13,811 (24.6%)	333,829 (22.5%)	
At risk	177,016 (11.5%)	6,320 (11.2%)	170,696 (11.5%)	
Distressed	223,512 (14.5%)	8,786 (15.6%)	214,726 (14.5%)	
Comorbidities				
Hypertension	518,821 (30.6%)	22,810 (37.1%)	496,011 (30.4%)	<0.001
Diabetes Mellitus	290,049 (17.1%)	14,776 (24.0%)	275,273 (16.8%)	<0.001
HIV	20,365 (1.2%)	1,988 (3.2%)	18,377 (1.1%)	<0.001
Obesity	377,732(22.3%)	22,884 (37.2%)	354,848 (21.7%)	<0.001
Dementia	121,226 (7.2%)	7,690 (12.5%)	113,536 (7.0%)	<0.001
Lifestyle behaviors				
Alcohol addiction	47,436 (1.3%)	453 (5.1%)	46,983 (1.3%)	<0.001
Smoking	312,256 (18.4%)	15,983 (26.0%)	296,273 (18.1%)	<0.001

CAP, Community-acquired pneumonia; DCI, Distressed Community Index. *p* < 0.05, statistical significance. Data are n (%) except otherwise stated.

Race/ethnicity differed significantly among the two groups. While 49.7% of patients with CAP identified as Non-Hispanic White and 36.0% as Non-Hispanic Black, patients who were Non-Hispanic White and Non-Hispanic Black in the No CAP group represented 53.4 and 33.4%, respectively. Among patients with CAP, 55.4% of the patients utilize Medicare, 23.5% use private insurance, and 2.4% are uninsured. Other variables such as income, comorbidities, and lifestyle behaviors also showed significant differences between the groups. Specifically, patients with comorbidities and lifestyle behaviors constituted a larger proportion of the CAP group than the No CAP group. Across the 5 strata of neighborhood economic deprivation, the DCI also varied significantly for both groups.

In Table 2, the logistic regression analysis presents the odds of CAP-related admissions, highlighting the associations with various significant risk factors. Age was noted as a critical factor in CAP-related admissions. Compared to the reference group (18–45 years), individuals aged 45–65 years had more than three times the odds (OR = 3.42, 95% CI: 3.32–3.52, *p* < 0.001) and those over 65 years had an even higher odds ratio (OR = 3.71, 95% CI: 3.57–3.85, *p* < 0.001). Female patients had a lower likelihood of CAP-related admissions compared to male patients (OR = 0.72, 95% CI: 0.71–0.74, *p* < 0.001). Racial and ethnic disparities were also evident; patients that identified as Black (OR = 1.25, 95% CI: 1.23–1.28, *p* < 0.001), Hispanic (OR = 2.11, 95% CI: 2.03–2.18, *p* < 0.001), Native American (OR = 1.61, 95% CI: 1.35–1.92, *p* < 0.001), and other races (OR = 1.40, 95% CI: 1.32–1.48, *p* < 0.001) had significantly higher odds of CAP-related admissions compared to White patients.

**Table 2:** Risk factors for admissions related to community acquired pneumonia, 2018–20.

Variable	Odds ratio	95% CI	*p*-value
Age (Years)			
18–45	Reference		
45–65	3.42	3.32–3.52	<0.001
>65	3.71	3.57–3.85	<0.001
Female (Ref. Male)	0.72	0.71–0.74	<0.001
Race/ethnicity			
White	Reference		
Black	1.25	1.23–1.28	<0.001
Hispanics	2.11	2.03–2.18	<0.001
Asian/Pacific Islander	1.20	1.13–1.26	<0.001
Native Americans	1.61	1.35–1.92	<0.001
Others	1.40	1.32–1.48	<0.001
Insurance			
Private	Reference		
Medicare	0.77	0.75–0.80	<0.001
Medicaid	0.97	0.94–0.99	0.03
Uninsured	1.84	1.73–1.95	<0.001
Others	0.50	0.38–0.64	<0.001
Income			
Quartile I	Reference		
Quartile II	1.11	1.05–1.15	<0.001
Quartile III	1.21	1.14–1.28	<0.001
Quartile IV	1.31	1.23–1.40	<0.001
DCI			
Prosperous	Reference		
Comfortable	1.09	1.06–1.12	<0.001
Mid-tier	1.25	1.20–1.29	<0.001
At risk	1.19	1.13–1.24	<0.001
Distressed	1.43	1.34–1.52	<0.001
Comorbidities			
Hypertension	1.03	1.01–1.04	<0.001
Diabetes Mellitus	1.06	1.04–1.08	<0.001
HIV	2.92	2.78–3.08	<0.001
Obesity	1.78	1.75–1.81	<0.001
Dementia	1.34	1.30–1.38	<0.001
Lifestyle behaviors			
Alcohol Abuse	0.67	0.64–0.71	<0.001
Smoking	1.09	1.07–1.12	<0.001

CAP, Community-acquired pneumonia; Ref., Reference; DCI, Distressed Community Index. *p* < 0.05, statistical significance.

Insurance type and income level also influenced CAP admissions. Compared to those with private insurance, uninsured patients had significantly higher odds of CAP-related admissions (OR = 1.84, 95% CI: 1.73–1.95, *p* < 0.001). Conversely, Medicare (OR = 0.77, 95% CI: 0.75–0.80, *p* < 0.001) and Medicaid patients (OR = 0.97, 95% CI: 0.94–0.99, *p* = 0.03) were less likely to be hospitalized on account of CAP. Income levels showed a gradient effect, with a higher likelihood of CAP admissions associated with higher income quartiles (Quartile II: OR = 1.11, 95% CI: 1.05–1.15, *p* < 0.001; Quartile III: OR = 1.21, 95% CI: 1.14–1.28, *p* < 0.001; Quartile IV: OR = 1.31, 95% CI: 1.23–1.40, *p* < 0.001). Comorbidities such as smoking, hypertension, diabetes mellitus, HIV, obesity, and dementia were also significant risk factors for CAP admissions, with obesity (OR = 1.78, 95% CI: 1.75–1.81, *p* < 0.001) and HIV (OR = 2.92, 95% CI: 2.78–3.08, *p* < 0.001) showing the highest odds ratios among these conditions.

**Figure 1:**
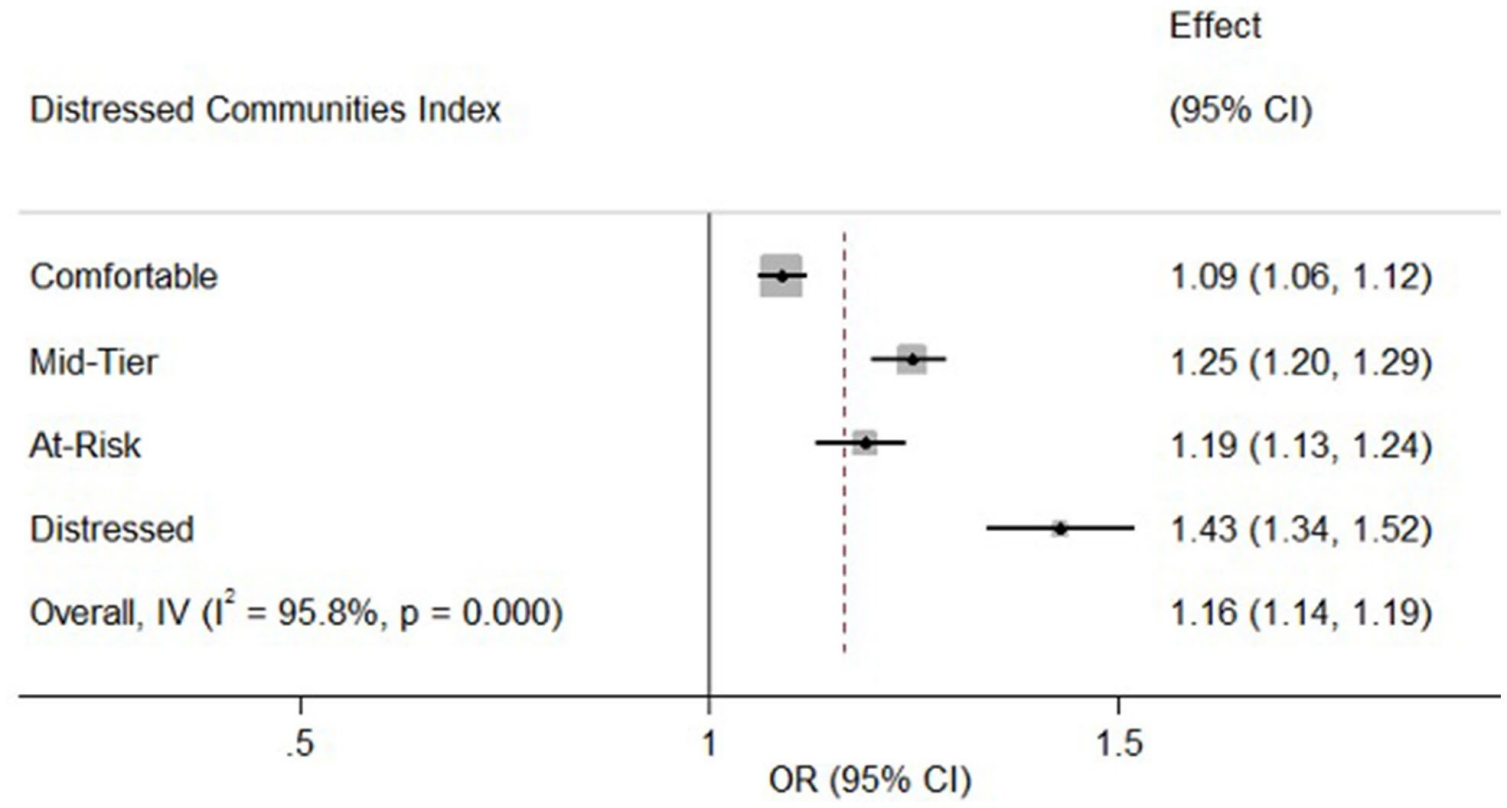
Association between neighborhood distress and CAP-related admissions. CI, Confidence Interval; IV, Inverse Variance; OR, Odds Ratio. The forest plot shows the association between the DCI and CAP-related admissions. OR (95% CI) indicate higher CAP risk with increasing community distress, compared to the richest neighborhoods (reference OR = 1.00). The vertical line represents the reference odds ratio (OR = 1.00) for residents of the richest neighborhoods. The overall effect estimate (OR = 1.16, 95% CI: 1.14–1.19) is also shown, indicating a significant association between higher community distress and increased risk of CAP-related admissions. The error bars represent the 95% CI for each OR, illustrating the precision of the estimates.

The analysis further revealed a significant association between DCI and CAP-related admissions. Patients from distressed communities had a notably higher odds ratio (OR = 1.43, 95% CI: 1.34–1.52, *p* < 0.001) for CAP admissions compared to those from prosperous communities. Similarly, those from mid-tier and at-risk communities also demonstrated increased odds of CAP admissions (OR = 1.25, 95% CI: 1.20–1.29, *p* < 0.001 and OR = 1.19, 95% CI: 1.13–1.24, *p* < 0.001 respectively). Even patients from comfortable communities showed a slight but significant increase in CAP risk (OR = 1.09, 95% CI: 1.06–1.12, *p* < 0.001). Figure 1 adequately illustrates the independent association between the DCI and CAP-related admissions by depicting the increased risk associated with various community distress levels compared to the reference group (residents of the poorest neighborhoods).

## Discussion

Our study importantly highlights the significant impact of socioeconomic conditions on the CAP-related hospitalizations in Maryland, particularly emphasizing the independent association of DCI with CAP-related admissions. We reported a striking 43% increase in CAP-related admissions among individuals living in the poorest neighborhoods compared to those residing prosperous neighborhoods. Our results align with previous studies that have shown similar associations between neighborhood poverty and increased rates of CAP-related admissions (33, 39).

Several potential pathways may explain the observed association. Individuals in distressed communities often experience multiple disadvantages, including limited access to healthcare, poor housing conditions, higher prevalence of chronic diseases, and greater exposure to environmental hazards (33, 40, 41). These factors collectively contribute to a higher risk of infections such as CAP. In addition, the stress associated with living in economically deprived areas can weaken immune responses, making residents more susceptible to infections (42, 43). The findings imply that addressing social determinants of health through policy interventions and community-level programs is crucial for reducing CAP incidence in impoverished neighborhoods.

Few studies have reported no significant association between neighborhood poverty and CAP-related admissions (44). These discrepancies could be due to regional variations in healthcare access, differences in study methodologies, or the presence of unmeasured confounding factors. Further efforts need to be expended to investigate these inconsistencies and to better understand the complex interplay between neighborhood socioeconomic status and health outcomes.

A critical factor influencing CAP-related admissions is age. Our analysis shows that individuals aged 45–65 years and those over 65 years had significantly higher odds of CAP-related admissions compared to the 18–45 age group. This age-related increase in CAP risk could be attributed to the natural decline in immune function with age, increased prevalence of comorbid conditions, and greater cumulative exposure to risk factors (41, 45, 46). These findings highlight the importance of targeted preventive measures, such as vaccination and early intervention, for older adults to mitigate their higher CAP risk.

Sex differences in CAP-related admissions were evident, with females exhibiting 28% lower odds of CAP-related admissions compared to males. This disparity may be as a result of biological differences, such as hormonal variations that affect immune responses, as well as behavioral factors, including healthcare-seeking behaviors and exposure to risk factors like smoking (47, 48). Understanding these sex-specific differences is crucial for developing tailored interventions to reduce CAP incidence among both men and women.

Our study also revealed significant racial and ethnic disparities in CAP-related admissions. Minority groups such as Blacks, Hispanics and Asian/Pacific Islander had higher odds of CAP-related admissions compared to Whites. These disparities likely reflect underlying socioeconomic inequalities, differences in healthcare access and utilization, and potential cultural influences on health behaviors. This pattern is seen in so many other conditions in the United States highlighting the systemic nature of significant racial disparities in health outcomes across a wide range of disease conditions (49). Efforts to address these disparities should focus on promoting access to healthcare, increasing cultural competence among healthcare providers, and implementing community-based interventions that address specific needs of these populations.

Insurance status emerged as a significant determinant of CAP-related admissions. Compared to individuals with private insurance, those on Medicare, Medicaid and other insurance had lower odds, while uninsured individuals had a substantially higher likelihood of CAP-related admissions. Individuals with Medicaid and Medicare had lower odds of CAP-related admissions which could be due to better access to preventive care and chronic disease management. In contrast, uninsured individuals were more likely to be admitted for CAP due to delayed healthcare seeking, lack of preventive care, poor chronic disease management, and socioeconomic disadvantages (40, 42). Addressing these disparities is crucial for improving health outcomes.

Income level showed a gradient association with CAP-related admissions, with higher odds observed in Quartile II (OR 1.11, 95% CI 1.05–1.15, *p* < 0.001), Quartile III (OR 1.21, 95% CI 1.14–1.28, *p* < 0.001), and Quartile IV (OR 1.31, 95% CI 1.23–1.40, *p* < 0.001) compared to Quartile I. Higher income was paradoxically associated with increased CAP-related admissions possibly because of advanced diagnostic practices and healthcare access leading to more frequent hospital admissions. These findings may be influenced by other factors not explored in this present study or complex interactions between the study variables and should therefore be interpreted with caution.

CAP-related admissions were also significantly by comorbid conditions and lifestyle behavior. Hypertension, diabetes mellitus, HIV, obesity, dementia and smoking were all associated with higher odds of CAP-related admissions (33, 50). These comorbidities likely contribute to increased susceptibility to infections and poorer health outcomes. Conversely, alcohol abuse was associated with lower odds of CAP-related admissions, which may be due to underreporting or complex interactions with other health behaviors and conditions.

## Limitations and strengths

This study has several limitations and strengths that should be noted. One major limitation is its observational design, which hinders our ability to establish causality between the identified risk factors and CAP-related admissions. In addition, while we controlled for several variables, there may be unmeasured confounders such as individual health behaviors and environmental exposures that were not fully accounted for. The reliance on administrative records for data accuracy is another limitation, as these records can contain errors or omissions that may affect the study’s findings. Moreover, socioeconomic status was measured at the neighborhood level rather than the individual level, which may not fully capture the nuances of personal socioeconomic conditions.

Despite these limitations, the study has significant strengths. The large and diverse sample size enhances the generalizability of the findings across different population groups. The comprehensive analysis of multiple socioeconomic and health-related factors provides a detailed understanding of the disparities in CAP-related admissions. This robust assessment allows for a nuanced examination of how distinct demographic groups are affected by CAP, informing targeted public health interventions. Additionally, the use of a well-validated measure, such as the DCI, strengthens the reliability of the socioeconomic status assessment.

## Conclusion

Our study found that individuals living in the poorest neighborhoods have significantly higher odds of CAP-related admissions compared to those in the wealthiest neighborhoods. This underscores the profound impact of neighborhood socioeconomic disparities on health outcomes. The public health implications highlight the urgent need for targeted interventions to improve access to healthcare and preventive services in socioeconomically disadvantaged areas. Further research is needed to explore the underlying mechanisms of these disparities and develop effective strategies to reduce the burden of CAP among vulnerable populations.

## Data availability statement

The data analyzed in this study is subject to the following licenses/restrictions: datasets are available with the authors and can be accessed by contacting the corresponding author. Requests to access these datasets should be directed to EO, eunice.odusanya@bison.howard.edu.

## Ethics statement

Ethical approval was not required for the study involving humans in accordance with the local legislation and institutional requirements. Written informed consent to participate in this study was not required from the participants or the participants' legal guardians/next of kin in accordance with the national legislation and the institutional requirements.

## Author contributions

OA: Conceptualization, Data curation, Formal analysis, Investigation, Methodology, Software, Validation, Visualization, Writing – original draft, Writing – review & editing. MF: Conceptualization, Data curation, Investigation, Project administration, Validation, Visualization, Writing – original draft, Writing – review & editing. EO: Conceptualization, Investigation, Project administration, Validation, Visualization, Writing – original draft, Writing – review & editing. TW: Conceptualization, Investigation, Project administration, Validation, Visualization, Writing – original draft, Writing – review & editing. OO: Project administration, Validation, Visualization, Writing – original draft, Writing – review & editing. MM: Supervision, Validation, Writing – review & editing, Writing – original draft. KH: Supervision, Validation, Writing – review & editing, Writing – original draft.
