# Supplementary material for: The relationship between neighborhood economic deprivation and community-acquired pneumonia related admissions in Maryland
Source: Front Public Health. 2024 Jul 18;12:1412671. doi: 10.3389/fpubh.2024.1412671 (PMC11291354; doi:10.3389/fpubh.2024.1412671)
Supplement: Supplementary file 1 [file Data_Sheet_1.docx]

**Supplementary File 1**

Covariates included in the Model, 2018-20

|  | Variable Name | Variable Levels |
| --- | --- | --- |
| Predisposing characteristics | Age (AGE)  Race (race2)  Gender (FEMALE) | 18-44; 45-64; 65+  Non-Hispanic White; Non-Hispanic Black; Hispanics; Non-Hispanic Asian/Pacific Islander: Non-Hispanic Native Americans; Others  Female; Male |
| Enabling Resources | Insurance (PAY)  Income (ZIPINC_QRTL)  DCI (DCI) | Medicare; Medicaid; Private; Uninsured; Others  Quartile I; Quartile II; Quartile III; Quartile IV  Prosperous; Comfortable; Mid-tier; At risk; Distressed |
| Need | Dementia  Alcohol Addiction  Obesity  Hypertension  Diabetes  HIV | Yes, No  Yes, No  Yes, No  Yes, No  Yes, No  Yes, No |

**Supplementary File 2**

ICD-10 Diagnosis code for community acquired pneumonia

J15.1 - Pneumonia due to Streptococcus pneumoniae

J18.1 - Lobar pneumonia, unspecified organism

J13 - Pneumonia due to Streptococcus pneumoniae

J18.9 - Pneumonia, unspecified organism

J18.0 - Bronchopneumonia, unspecified organism

J15.9 - Pneumonia due to unidentified organism

J18.2 - Hypostatic pneumonia, unspecified organism

J18.8 - Other pneumonia, organism unspecified

J16.8 - Pneumonia due to other specified infectious organisms

J18.9 - Pneumonia, unspecified organism

I10_dx`c'=="A0222"|I10_DX`c'=="A202" |I10_DX`c'=="A212" |I10_DX`c'=="A221"

I10_DX`c'=="A310" |I10_DX`c'=="A420" |I10_DX`c'=="B012" |I10_DX`c'=="B052"

I10_dx`c'=="J17" |I10_DX`c'=="B371" |I10_DX`c'=="B380" |I10_DX`c'=="B381"

I10_DX`c'=="B392" |I10_DX`c'=="B583" |I10_DX`c'=="B59" |I10_DX`c'=="J120"

I10_DX`c'=="J121" |I10_DX`c'=="J122"

I10_dx`c'=="J1281"|I10_DX`c'=="J1289" |I10_DX`c'=="J129" |I10_DX`c'=="J13"

I10_DX`c'=="J181" |I10_DX`c'=="J150" |I10_DX`c'=="J151" |I10_DX`c'=="J14"

I10_DX`c'=="J154" |I10_DX`c'=="J153"

I10_dx`c'=="J1520"|I10_DX`c'=="J15211"|I10_DX`c'=="J15212" |I10_DX`c'=="J1529"

I10_DX`c'=="J155" |I10_DX`c'=="J156" |I10_DX`c'=="A481" |I10_DX`c'=="J158"

I10_DX`c'=="J159"

I10_dx`c'=="J157" |I10_DX`c'=="J160" |I10_DX`c'=="J168" |I10_DX`c'=="B250"

I10_DX`c'=="A3791"|I10_DX`c'=="A221" |I10_DX`c'=="B440" |I10_DX`c'=="J180"

I10_DX`c'=="189" |I10_DX`c'=="J851"
